# Assessing the clinical severity of the Omicron variant in the Western Cape Province, South Africa, using the diagnostic PCR proxy marker of RdRp target delay to distinguish between Omicron and Delta infections – a survival analysis

**DOI:** 10.1016/j.ijid.2022.02.051

**Published:** 2022-05

**Authors:** Hannah Hussey, Mary-Ann Davies, Alexa Heekes, Carolyn Williamson, Ziyaad Valley-Omar, Diana Hardie, Stephen Korsman, Deelan Doolabh, Wolfgang Preiser, Tongai Maponga, Arash Iranzadeh, Sean Wasserman, Linda Boloko, Greg Symons, Peter Raubenheimer, Arifa Parker, Neshaad Schrueder, Wesley Solomon, Petro Rousseau, Nicole Wolter, Waasila Jassat, Cheryl Cohen, Richard Lessells, Robert J Wilkinson, Andrew Boulle, Nei-yuan Hsiao

**Affiliations:** 1 Health Intelligence, Western Cape Government: Health, South Africa; 2 Division of Public Health Medicine, School of Public Health and Family Medicine, University of Cape Town, South Africa; 3 Centre for Infectious Disease Epidemiology and Research, School of Public Health and Family Medicine, University of Cape Town, South Africa; 4 Division of Medical Virology, University of Cape Town, Cape Town, Western Cape, South Africa; 5 National Health Laboratory Service, South Africa; 6 Wellcome Centre for Infectious Diseases Research in Africa, Institute of Infectious Disease and Molecular Medicine, University of Cape Town, South Africa; 7 Division of Medical Virology, University of Stellenbosch, South Africa; 8 Division of Infectious Diseases and HIV Medicine, Department of Medicine, University of Cape Town, South Africa; 9 Department of Medicine, Groote Schuur Hospital, University of Cape Town, South Africa; 10 Department of Medicine, Tygerberg Hospital, Stellenbosch University, South Africa; 11 National Department of Health, South Africa; 12 National Institute for Communicable Diseases of the National Health Laboratory Service, Johannesburg, South Africa; 13 School of Pathology, University of the Witwatersrand, Johannesburg, South Africa; 14 School of Public Health, Faculty of Health Sciences, University of the Witwatersrand, Johannesburg, South Africa; 15 KwaZulu-Natal Research, Innovation & Sequencing Platform, University of KwaZulu-Natal, Durban, South Africa; 16 The Francis Crick Institute, Midland Road, London, NW1 1AT, UK; 17 Department of Infectious Diseases, Imperial College London, W12 0NN, UK

**Keywords:** SARS-CoV-2, Omicron variant, clinical severity, South Africa, RdRp target delay

## Abstract

**Background:**

At present, it is unclear whether the extent of reduced risk of severe disease seen with SARS-Cov-2 Omicron variant infection is caused by a decrease in variant virulence or by higher levels of population immunity.

**Methods:**

RdRp target delay (RTD) in the Seegene Allplex^TM^ 2019-nCoV PCR assay is a proxy marker for the Delta variant. The absence of this proxy marker in the transition period was used to identify suspected Omicron infections.

Cox regression was performed for the outcome of hospital admission in those who tested positive for SARS-CoV-2 on the Seegene Allplex^TM^ assay from November 1 to December 14, 2021 in the Western Cape Province, South Africa, in the public sector. Adjustments were made for vaccination status and prior diagnosis of infection.

**Results:**

A total of 150 cases with RTD and 1486 cases without RTD were included. Cases without RTD had a lower hazard of admission (adjusted hazard ratio [aHR], 0.56; 95% confidence interval [CI], 0.34-0.91). Complete vaccination was protective against admission, with an aHR of 0.45 (95% CI, 0.26-0.77).

**Conclusion:**

Omicron has resulted in a lower risk of hospital admission compared with contemporaneous Delta infection, when using the proxy marker of RTD. Under-ascertainment of reinfections with an immune escape variant remains a challenge to accurately assessing variant virulence.

## Highlights

• Lower risk of admission with Omicron compared with contemporaneous Delta cases
• Analysis adjusted for vaccination status and prior diagnosed infection
• Compares contemporaneous cases, which is more robust than other South African studies
• Only the second study from a low middle income country assessing Omicron with contemporaneous cases
• Shows ongoing utility of novel proxy marker (RdRp target delay on Seegene Allplex assay)

## Background

With the rapid global spread of the Omicron (B.1.1.529) SARS-CoV-2 variant of concern (VOC), understanding the clinical implications of this new VOC is critical (Viana et al., 2022). Emerging data from both South Africa and the United Kingdom suggest that this variant is associated with a reduced risk of severe disease (Abdullah et al., 2021, Ferguson et al., 2022; Jassat et al., 2021; Maslo et al., 2021; Sheikh et al., 2021; Wolter et al., 2021). The extent to which this reflects a difference in the inherent virulence of Omicron, or just higher levels of population immunity due to previous infection and/or vaccination, is currently not clear.

In November 2021 in the Western Cape Province, South Africa, following a period of very low incidence, Omicron rapidly replaced Delta (B.1.617.2) as the dominant variant and drove the fourth wave of infections. Omicron is known to result in S-gene target failure (SGTF) on the Thermo Fisher TaqPath^TM^ PCR assay (Scott et al., 2021). Unfortunately, too little routine diagnostic testing is done using this assay in the Western Cape National Health Laboratory Service (NHLS) to power an SGTF analysis.

RdRp target delay (RTD) is a proxy marker for the Delta variant on routine diagnostic testing with the Seegene Allplex^TM^ 2019-nCoV PCR assay, similar in concept to SGTF. RTD has a 93.6% sensitivity and 89.7% specificity in detecting the Delta variant when compared to genomic sequencing (Valley-Omar et al., 2022). This proxy marker has previously been used to successfully assess the association of the Delta variant and mortality in the third wave (Hussey et al., 2021).

**Figure 1:**
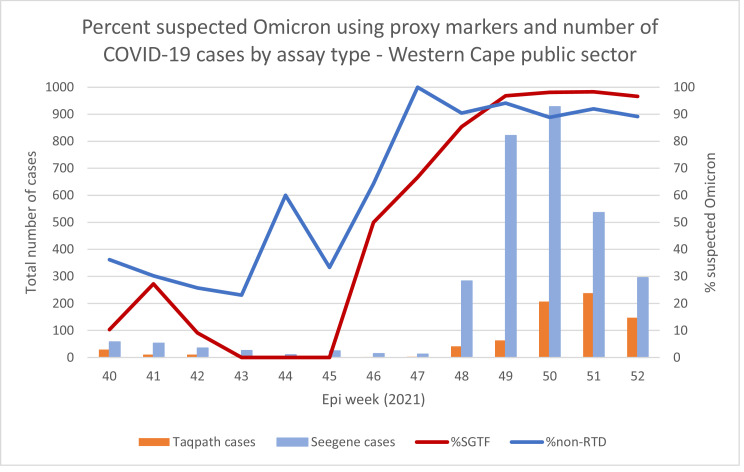
Percentage of suspected Omicron using the proxy markers of S-gene target failure in Thermo Fisher TaqPath^TM^ cases and non-RdRp target delay in Seegene Allplex^TM^ assay cases in Western Cape Province public sector adults.

As the Seegene Allplex^TM^ assay is widely used by the Western Cape NHLS, and as Omicron rapidly displaced the Delta variant with minimal other variants detected, the absence of RTD in Seegene Allplex^TM^ cases can be used to identify likely Omicron infections during the replacement period. The absence of RTD tracks well with SGTF in the province (Figure 1), as well as genomic sequenced data (Network for Genomic Surveillance in South Africa (NGS-SA), 2021; Viana et al., 2022). We used this proxy marker to assess the clinical severity associated with Omicron infection.

## Methods

RTD is defined as a difference in cycle threshold value of RdRp gene target–E gene target >3.5 in the Seegene Allplex^TM^ 2019-nCoV PCR assay (Valley-Omar et al., 2022). Cases diagnosed using this assay have previously been found to be representative of cases diagnosed by any PCR assay in the Western Cape Province public sector (Hussey et al., 2021).

We included all COVID-19 cases, aged 15 years and older, diagnosed with the Seegene Allplex^TM^ 2019-nCoV diagnostic PCR assay in the Western Cape Province public sector from November 1 to December 14, 2021, a period when both Delta and Omicron were cocirculating and other lineages were negligible (Network for Genomic Surveillance in South Africa (NGS-SA), 2021; Viana et al., 2022). Follow-up ended on January 6, 2022. Children were excluded from this analysis as their testing and admission patterns are different from those of adults.

The Western Cape Provincial Health Data Centre (PHDC) collates all available electronic health data on public sector patients, including COVID-19 test results, comorbidities, and hospital admissions. If an individual tested positive for SARS-CoV-2 14 days before admission, or 7 days after the date of admission, and was not admitted to a specialized psychiatric and rehabilitation facility, this was defined as COVID-19–related hospital admission.

We undertook a survival analysis using Cox regression, assessing time from date of diagnosis to date of hospital admission, which was our outcome of interest. Those whose date of admission was on or before the date of diagnosis were assigned 1 day of follow-up. Intensive Care Unit (ICU) admission or mortality were not assessed as too few of these severe outcomes had occurred. We adjusted for age, sex, known comorbidities, prior diagnosed infection (2 positive COVID-19 tests more than 90 days apart), and vaccination status at time of diagnosis. Vaccination status was determined by linking COVID-19 cases with the national Electronic Vaccination Data System through national identifiers in the PHDC. Complete vaccination was defined as ≥28 days after vaccination with Janssen/Johnson & Johnson (Ad26.COV2.S), or ≥14 days after the second dose of Pfizer–BioNTech (BNT162b2). Patients were deemed partially vaccinated from the day after their (first) vaccine dose until meeting criteria for complete vaccination. Analyses were conducted using Stata 13.1 (StataCorp).

## Results

A total of 1636 cases were tested on the Seegene Allplex ^TM^ assay during the study period and included in this analysis, representing 14% of all cases diagnosed and 24% of all cases diagnosed by PCR testing in the public sector at that time. Of these, 150 cases were with RTD (proxy for Delta) and 1486 cases were without RTD (proxy for Omicron/non-Delta). Patient demographic characteristics and comorbidities were similar in both groups (Table 1). The median age in both groups was 33 years (interquartile range [IQR], 25-44 in those with RTD; IQR, 26-44 in those without). The proportion of cases that were fully vaccinated in both groups was also similar, although more infections following partial vaccination were seen in those without RTD.

**Table 1:** Characteristics of included cases, according to presence or absence of RdRp target delay, and adjusted hazard ratios for the outcome of admission in adults in the Western Cape Province public sector, November 1 to December 14, 2021.

		RTD present	RTD absent	Cox regression for outcome of admission	
	n	150	1486	1636 (122 admissions)	
		n (%)	n (%)	adjusted HR	95% CI
RTD	**Present ("Delta")**			**Ref**	
	**Absent ("Omicron")**			**0.56**	0.34; 0.91
Sex	**Female**	80 (53.3%)	860 (57.9%)	**Ref**	
	**Male**	70 (46.7%)	626 (42.1%)	**1.03**	0.71; 1.48
					
Age category	**15-29 years**	57 (38%)	559 (37.6%)	**Ref**	
	**30-39 years**	43 (28.7%)	447 (30.1%)	**0.92**	0.56; 1.50
	**40-49 years**	21 (14%)	216 (14.5%)	**1.12**	0.62; 2.02
	**50-59 years**	15 (10%)	166 (11.2%)	**0.91**	0.46; 1.80
	**60-69 years**	6 (4%)	60 (4%)	**1.60**	0.73; 3.49
	**≥70 years**	8 (5.3%)	38 (2.6%)	**1.27**	0.56; 2.89
Comorbidity§	**HIV positive**	21 (14%)	142 (9.6%)	**1.29**	0.72; 2.30
	**Diabetes**	7 (4.7%)	77 (5.2%)	**1.30**	0.68; 2.46
	4 (2.7%)	17 (1.1%)	**5.15**		
	**Hypertension**	19 (12.7%)	149 (10%)	**1.78**	0.99; 3.19
	4 (2.7%)	23 (1.6%)		**4.46**	
	**COPD / Asthma**	6 (4%)	79 (5.3%)	**2.26**	1.29; 3.96
Prior documented infection	**None**	134 (89.3%)	1313 (88.4%)	**Ref**	
	**Yes**	16 (10.7%)	173 (11.6%)	**0.00**	
Vaccination†	**None**	114 (76%)	1081 (72.7%)	**Ref**	
	**Partial**	3 (2%)	93 (6.3%)	**1.22**	0.63; 2.36
	**Complete**	33 (22%)	312 (21%)	**0.45**	0.26; 0.77

§ The reference group for the aHR here is the absence of that specific comorbidity.

† Fully vaccinated was defined as ≥28 days post-vaccination with Janssen/Johnson & Johnson or ≥14 days post second dose of Pfizer–BioNTech. Patients were deemed partially vaccinated from the day after their (first) vaccine dose until meeting criteria for complete vaccination.

The proportion of cases with a documented reinfection was 11% for both those with and without RTD. By using a stricter definition of RTD (ie, requiring a larger difference in cycle threshold values), a similar proportion of RTD cases that had documented reinfections was found (Supplementary Table 1).

There were 21 cases (14%) admitted to hospital among those with RTD, whereas 101 (6.8%) were admitted among those without RTD. Amongst those not admitted, 189 (12.5%) had a documented previous infection, whereas amongst those admitted, none had a documented previous infection, and so we could not calculate the extent of protection from prior diagnosed infection against admission, although there appears to be benefit. The cases without RTD (ie, suspected Omicron cases) had a lower hazard of admission (adjusted hazard ratio [aHR], 0.56; 95% confidence interval [CI], 0.34-0.91) (Table 1) than suspected Delta cases after adjusting for age, sex, prior diagnosed infection, vaccination status at time of diagnosis, and known comorbidities. Complete vaccination was protective against admission, with an aHR of 0.45 (95% CI, 0.26-0.77).

The proportional hazards assumption was tested and found to have held for each variable and the model as a whole (Supplementary Table 2).

## Discussion

Using the proxy marker of RTD absence, suspected Omicron cases were associated with a lower risk of hospital admission than that of contemporaneous suspected Delta cases.

A study from the South African National Institute for Communicable Diseases found that using SGTF, suspected Omicron cases had lower odds of being admitted to hospital compared with non-SGTF infections (adjusted odds ratio [aOR], 0.2; 95% CI, 0.1-0.3) (Wolter et al., 2021). However, this analysis was not able to adjust for vaccination status at time of diagnosis, potentially explaining the greater reduction in observed disease severity compared with our study. Similar contemporaneous SGTF studies from the United Kingdom have also found that SGTF was associated with a lower risk of hospitalization, with an adjusted observed/expected ratio of 0.32 (95% CI, 0.19-0.52) (Sheikh et al., 2021) and a 40% to 45% reduction in risk of admission, which is similar to our result (Ferguson et al., n.d.).

Several studies have also found less severe disease in the fourth Omicron-driven wave compared with the third wave caused by Delta in South Africa (Abdullah et al., 2021; Jassat et al., 2021; Maslo et al., 2021). However, as they are comparing noncontemporaneous cases, it is difficult to account for the effect of the vaccination program, which started too late to provide significant protection against the third wave (Mathieu et al., 2021). In addition, more individuals had some immunity from a previous infection at the start of the fourth wave compared with the third.

Anecdotally, this fourth wave has resulted in a relatively large proportion of admissions where COVID-19 was incidentally diagnosed (Abdullah et al., 2021). It is unfortunately not possible to ascertain the primary indication for admission from our routine data and we were unable to assess severe outcomes because of the very small numbers of patients being tested on the Seegene Allplex^TM^ assay, who were recorded as having steroids prescribed electronically, being admitted to ICU, or dying. The fact that full vaccination still provided substantial protection against admission, even with the contamination of incidental admissions, suggests that vaccination provides very strong protection against admission in the face of the Omicron variant, and that some of the admissions were likely due to COVID-19 disease. Vaccination itself might also be a proxy marker for higher socioeconomic status, access to health care, or good health seeking behaviour, such as adherence to chronic medications, and as such might confer some protection against hospital admissions, irrespective of COVID-19.

Omicron is associated with an increased risk of reinfections (Pulliam et al., 2021). Previous infection in itself is protective against severe disease (Milne et al., 2021). In this analysis, no cases with a documented reinfection required hospital admission. However, seroprevalence studies indicate only a small fraction of total COVID-19 cases in the Western Cape Province are laboratory-confirmed (Hsiao et al., 2020), particularly when there have been testing restrictions during epidemic surges. Under-ascertainment of prior diagnosed infection is therefore likely, and this could lead to a considerably biased observation of disease severity for an immune escape variant when compared with a variant that is not associated with increased  reinfection. The extent of this residual confounding, ie, the contribution of this under-ascertainment of reinfections to the milder disease severity seen with Omicron, is thus still uncertain in our context of very high rates of unconfirmed prior infection. At the same time, if we are diagnosing a smaller proportion of Omicron cases by missing the more mild or asymptomatic infections, or if the Omicron variant results in more incidental hospital admissions and outcome misclassification, this could bias our findings in the opposite direction, and falsely elevate the disease severity seen with this variant.

An additional limitation to this study is the low number of SARS-CoV-2 infections during the Delta to Omicron transition period, particularly in the public sector, to which this analysis was restricted, as well as the increasing use of SARS-CoV-2 rapid antigen tests (used in diagnosing 42% of cases in the study period) from which the variant proxy markers cannot be calculated. We also used a proxy marker for Omicron and not whole-genome sequencing, as only limited sequencing is feasible in our setting. A proxy marker on routine diagnostic testing is likely to result in some variant misclassification, which is evidenced by the high rate of reinfections seen in RTD cases. Attempts to narrow the definition of RTD were not able to resolve this. In addition, this analysis, like many others, only compares disease severity between Omicron and Delta. Little is known of the severity of Omicron when compared with the ancestral strain and other non-Delta VOCs.

## Conclusion

Omicron has resulted in a lower risk of hospital admission when compared with contemporaneous Delta cases using the proxy marker of RTD in the Western Cape Province. Vaccination and documented previous infection are highly protective against hospital admission. Under-ascertainment of reinfections with an immune escape variant like Omicron remains a challenge to accurately assessing variant virulence.

## Appendix Supplementary materials

Image, application 1

Image, application 2

Image, application 3
