# Supplementary figures and images for: Assessing the clinical severity of the Omicron variant in the Western Cape Province, South Africa, using the diagnostic PCR proxy marker of RdRp target delay to distinguish between Omicron and Delta infections – a survival analysis
Source: Int J Infect Dis. 2022 May;118:150–4. doi: 10.1016/j.ijid.2022.02.051 (PMC8882068; doi:10.1016/j.ijid.2022.02.051)

1.
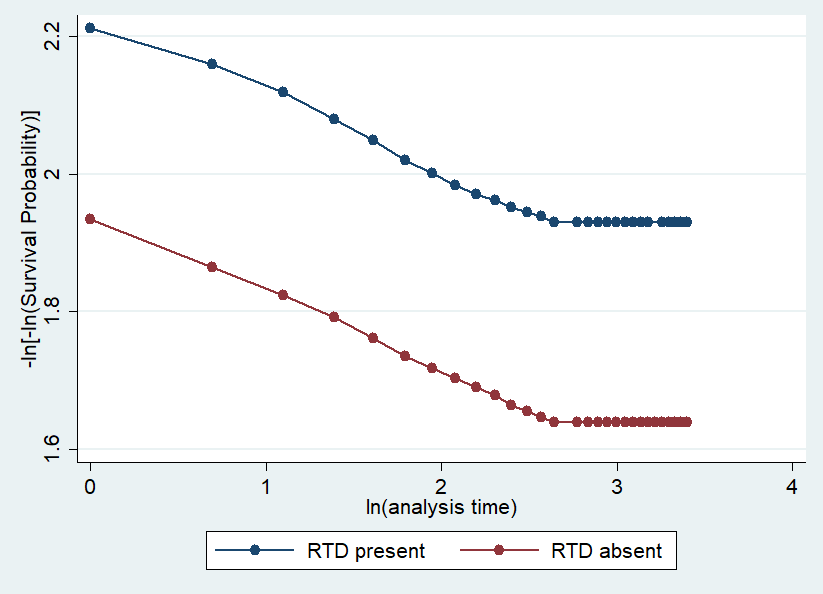

2.
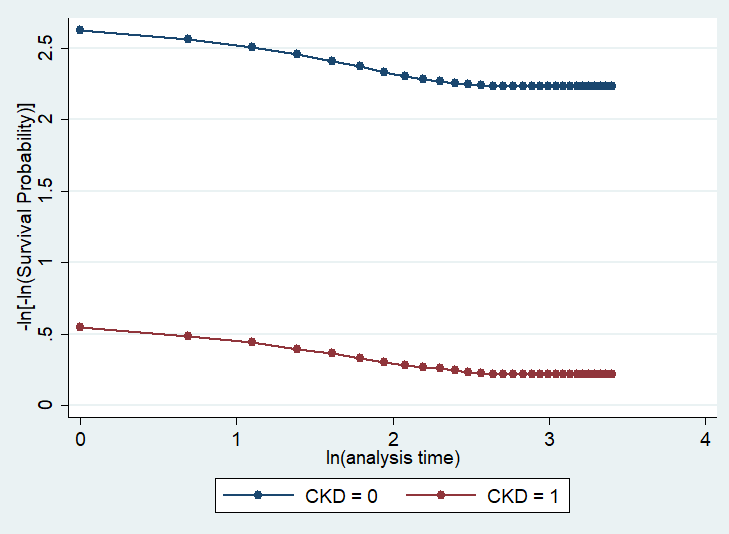

3.
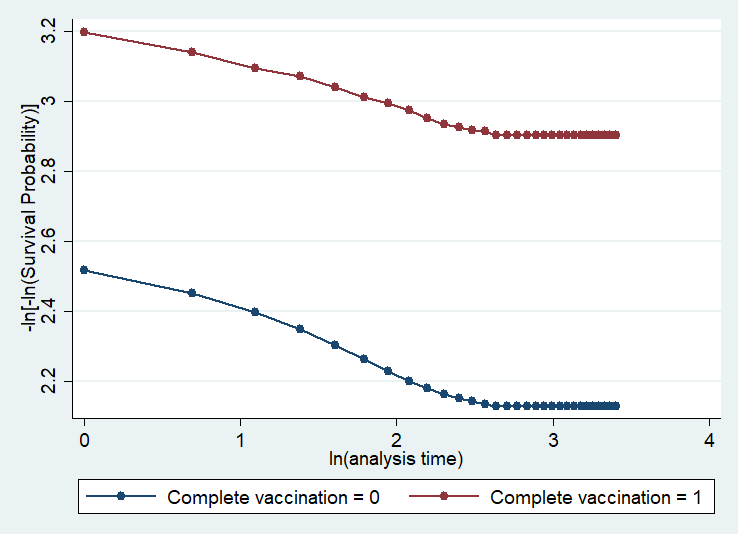


**Supplementary Figure 1: Log-log plots for the covariates of (a) RTD, (b) CKD and (c) complete vaccination.**

Supplement: Supplementary file 1 [file mmc1.docx]
